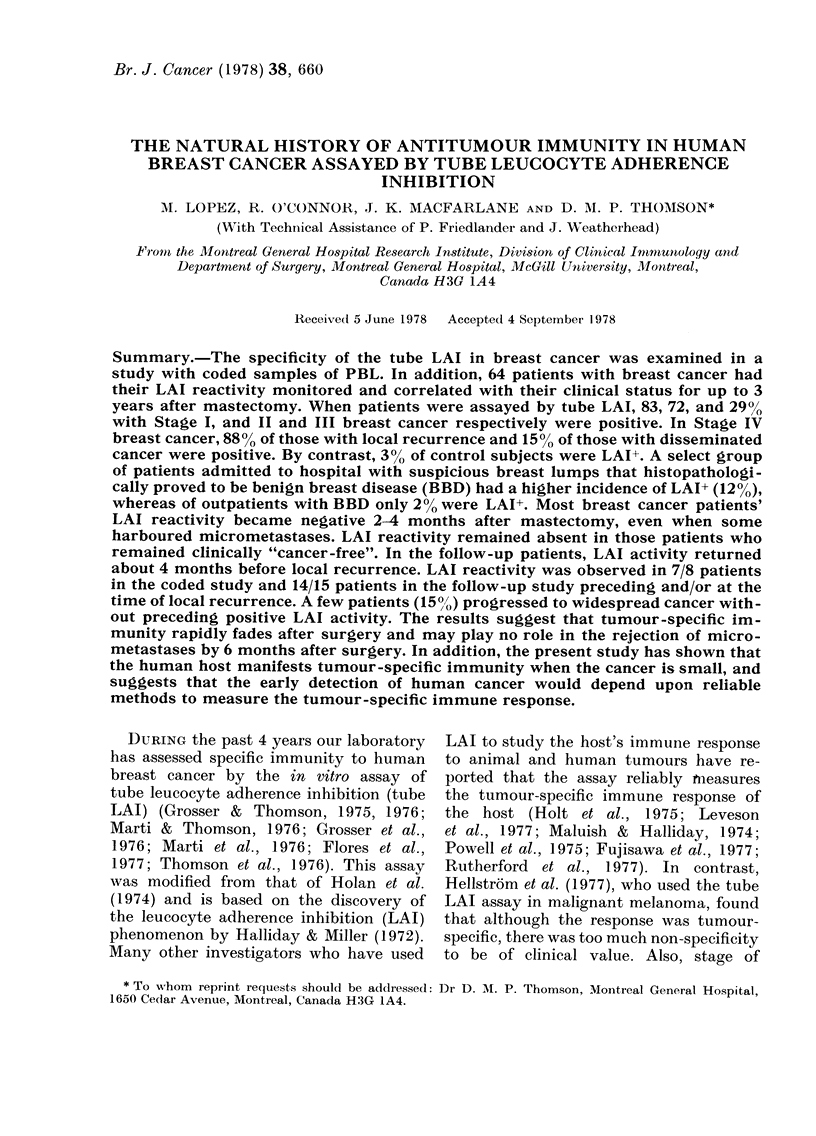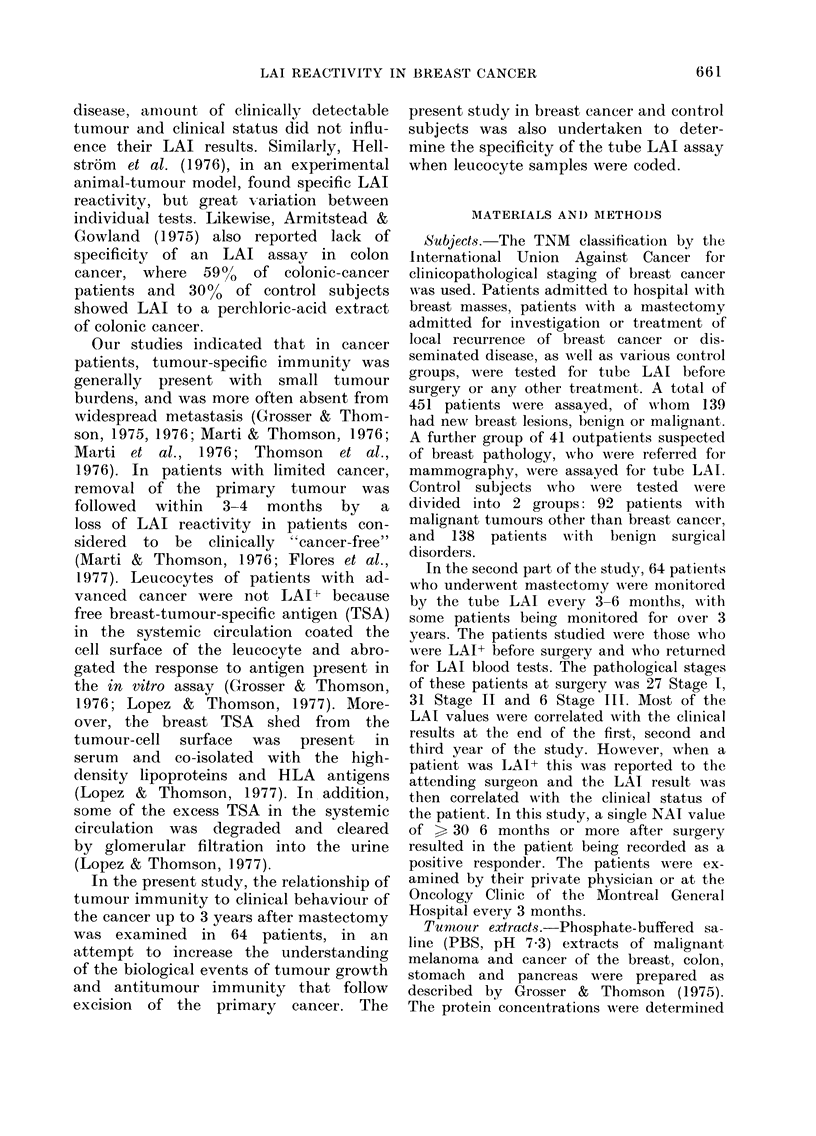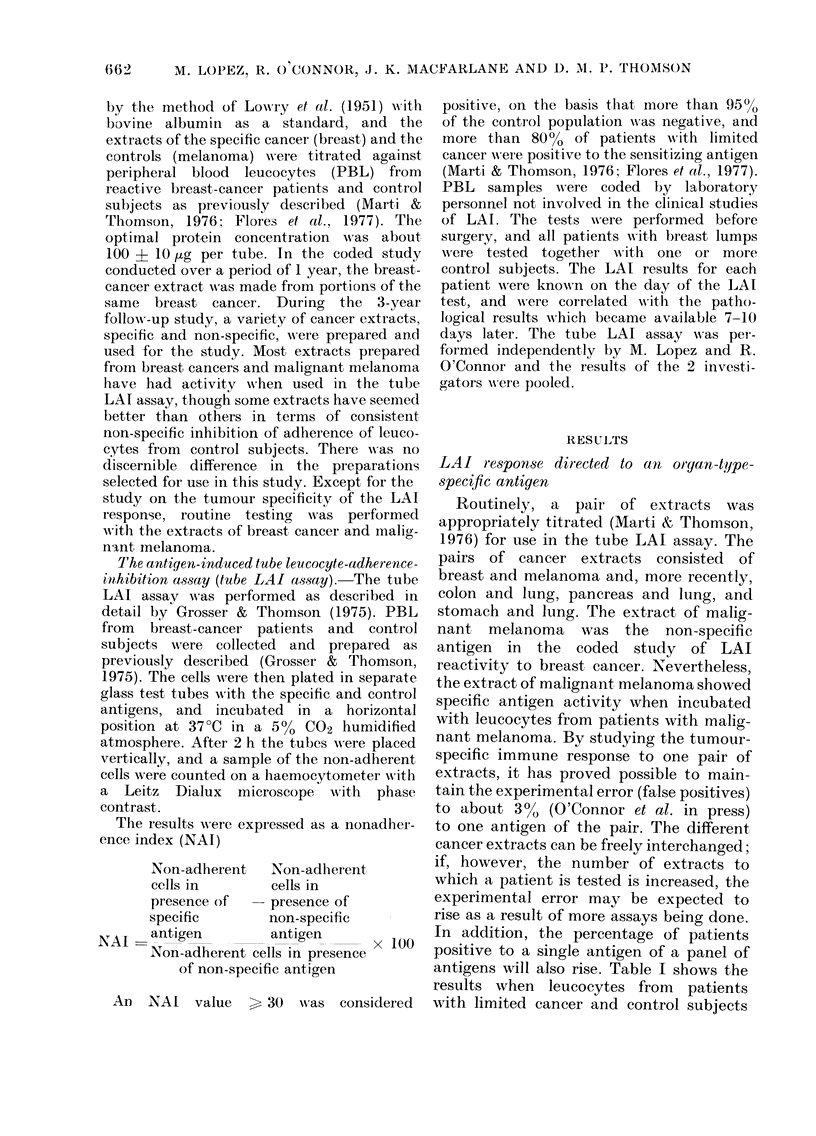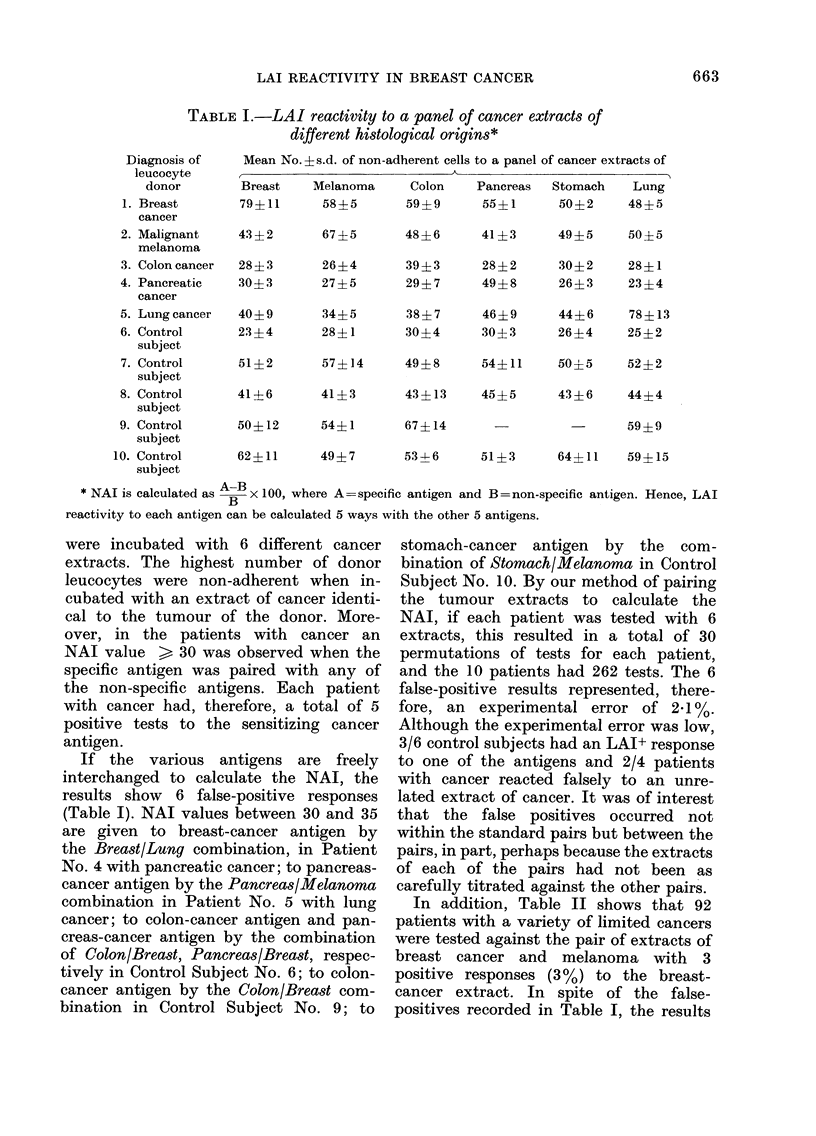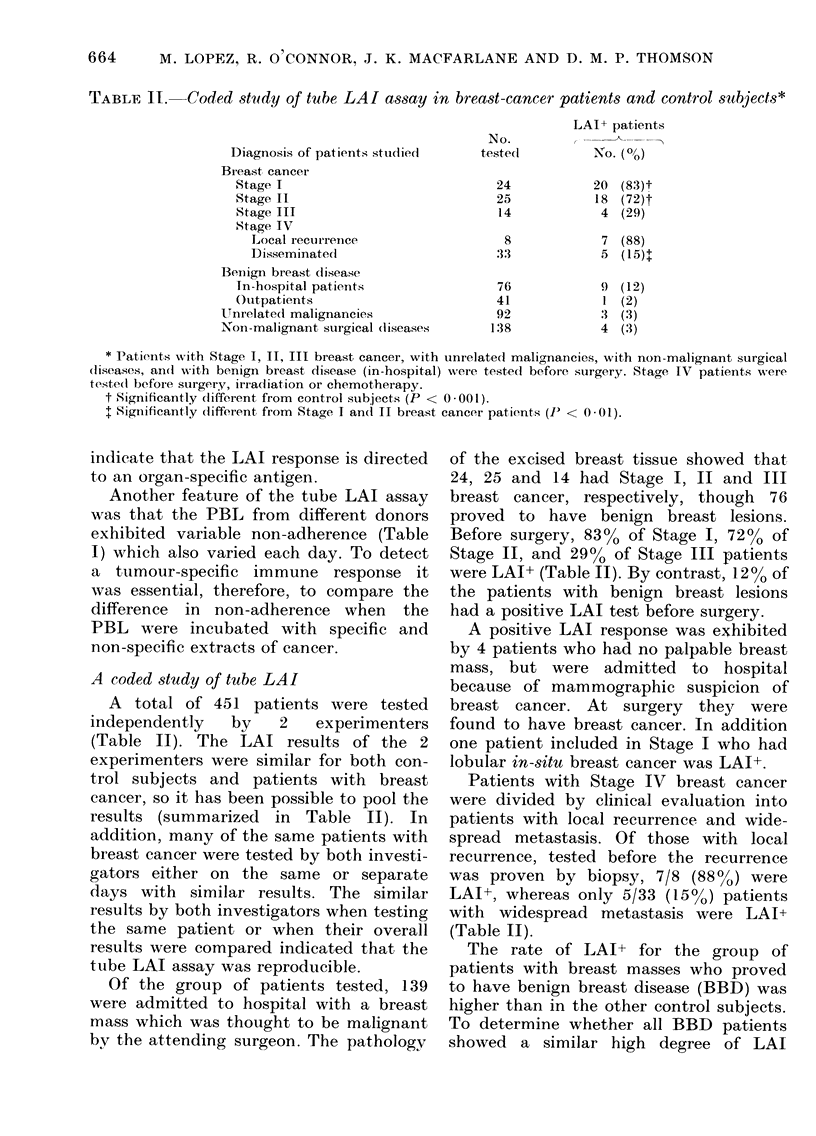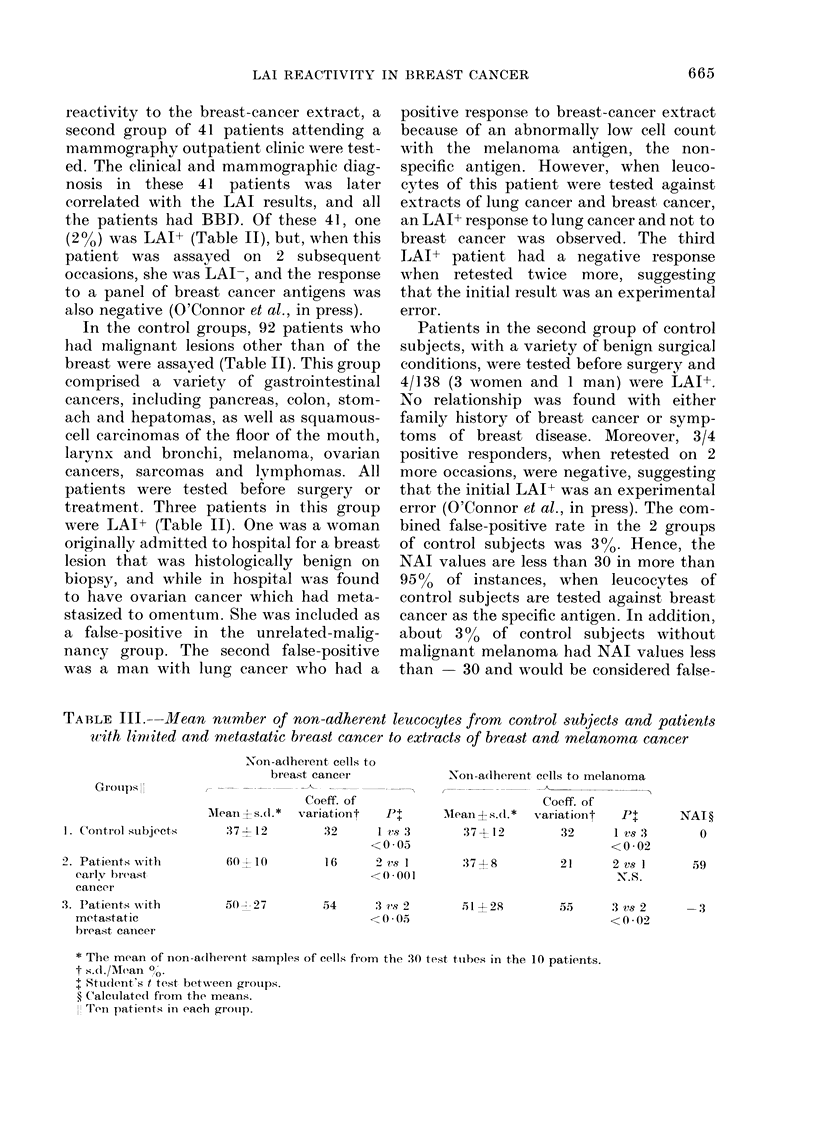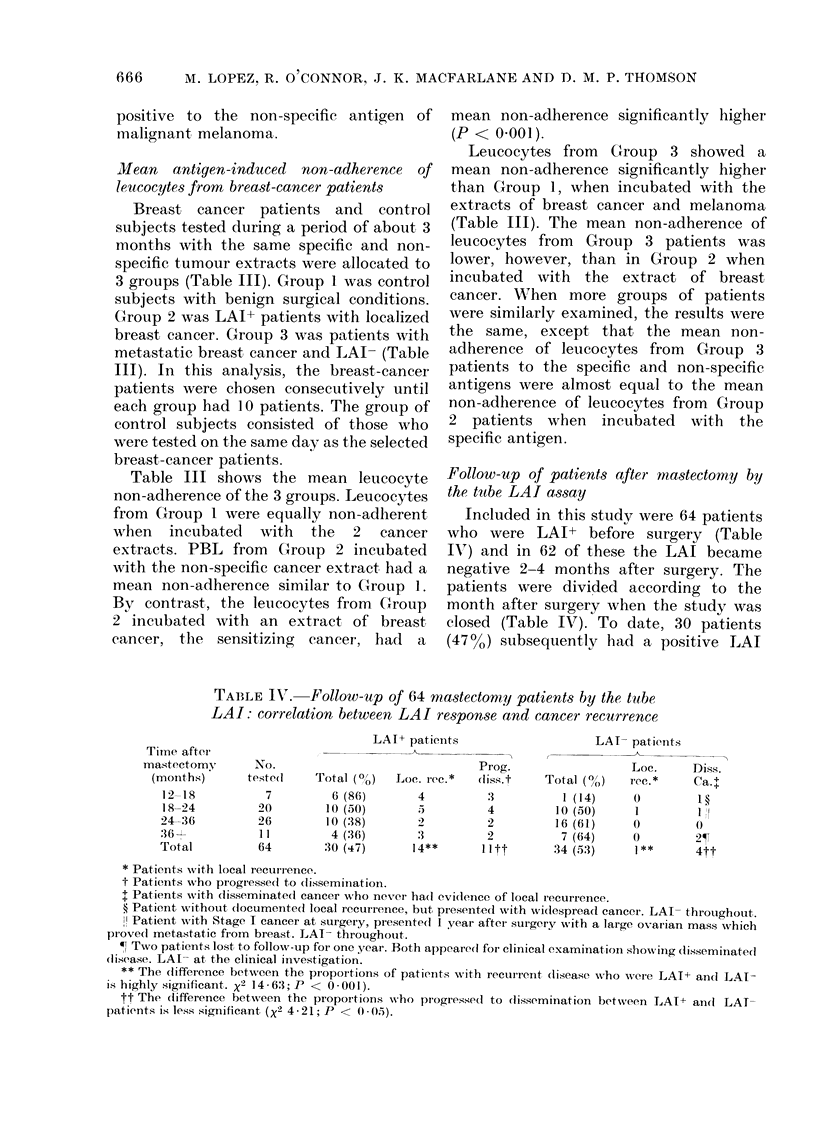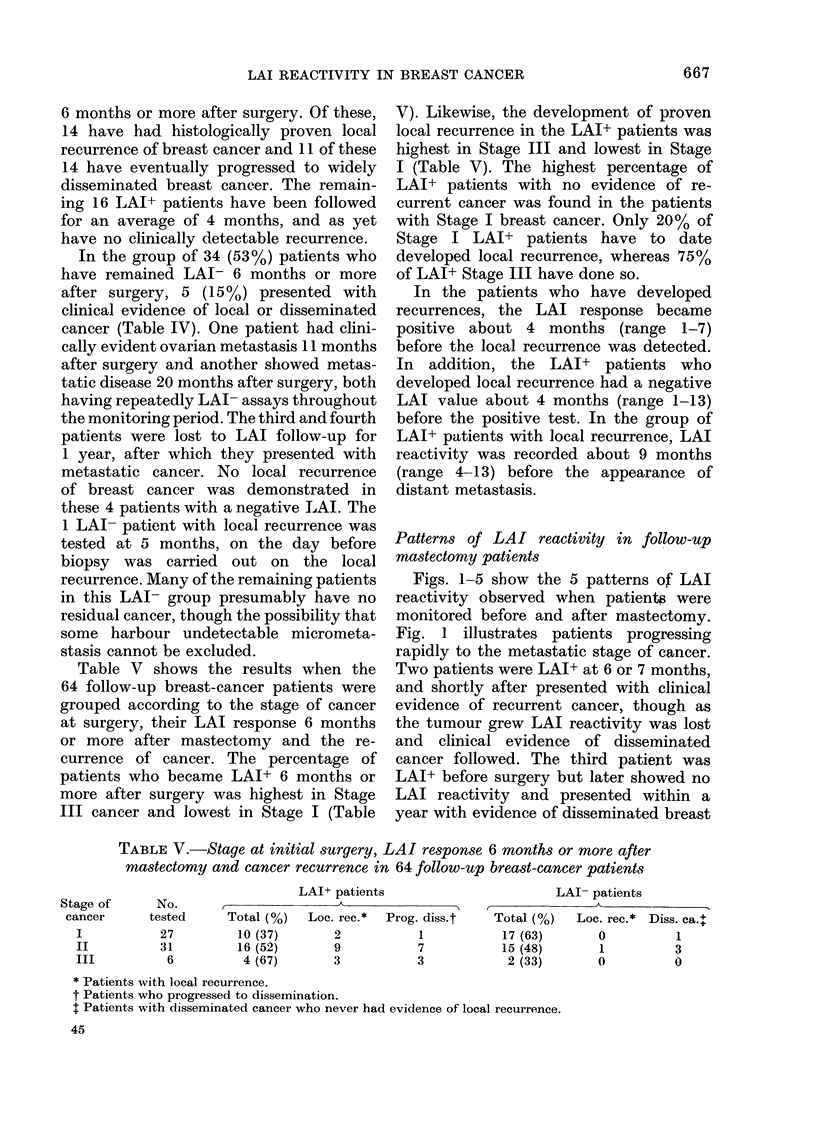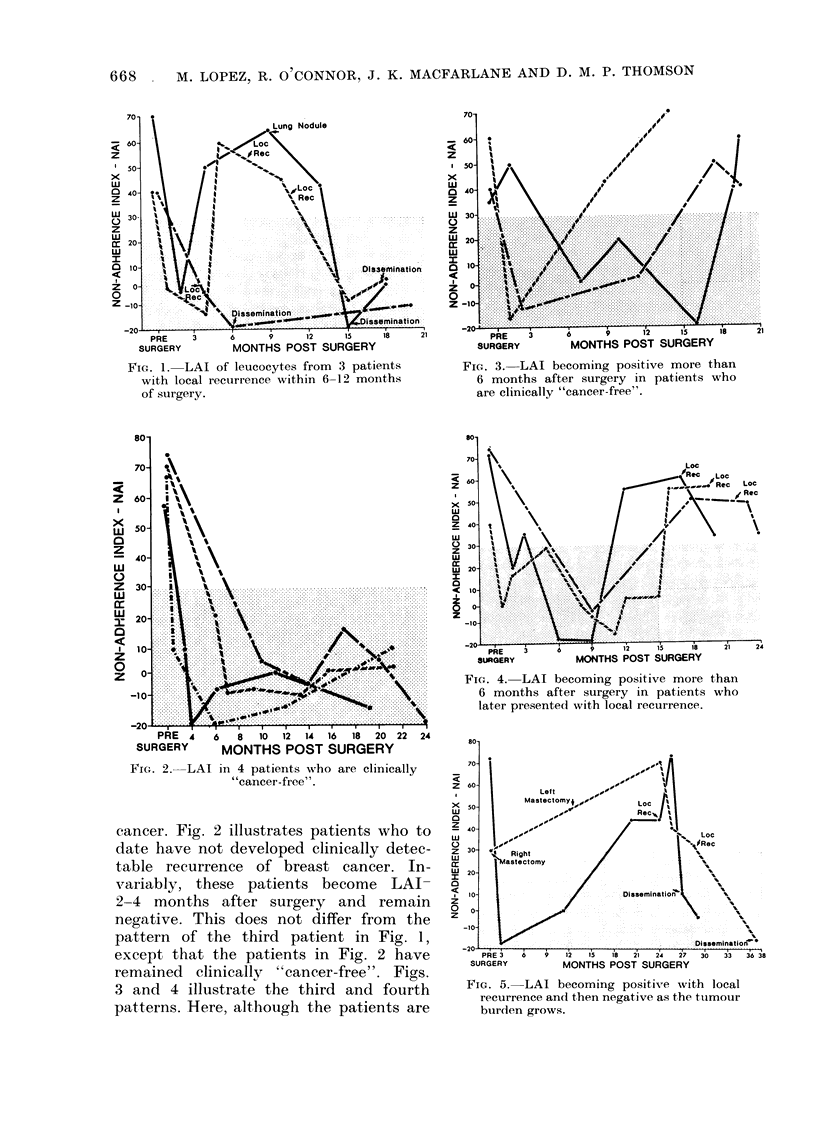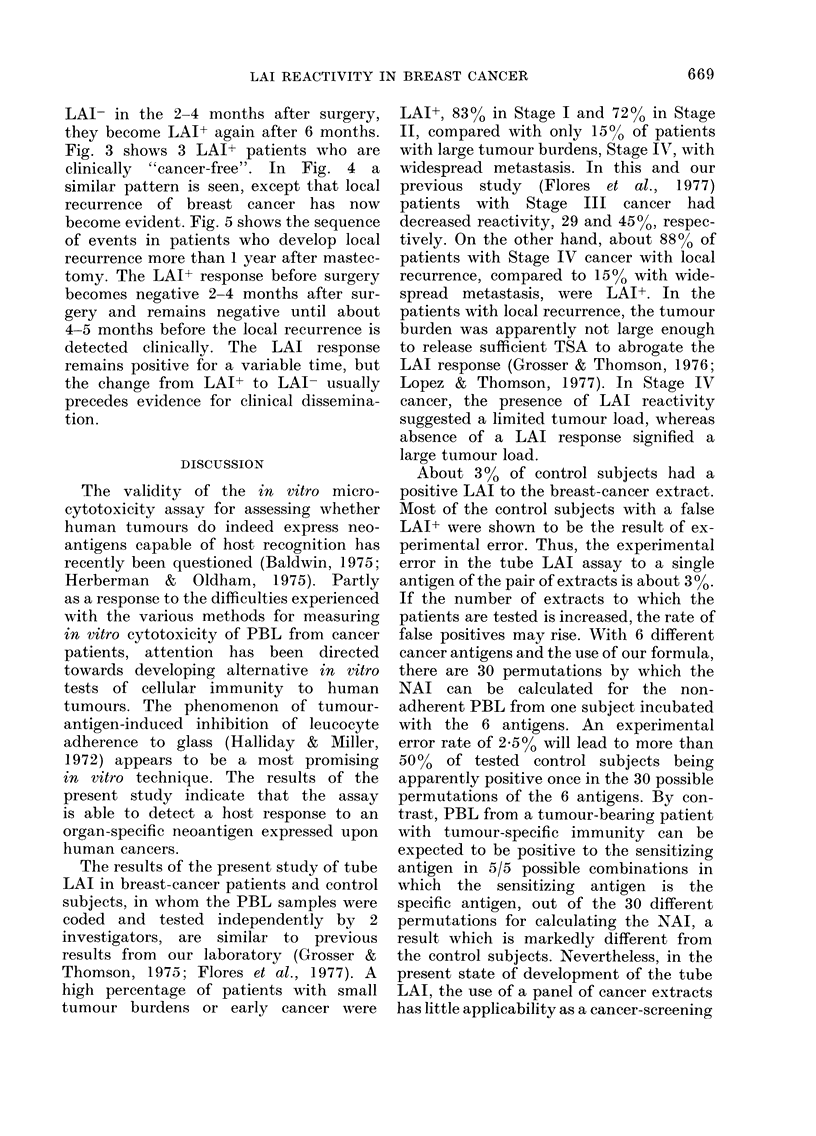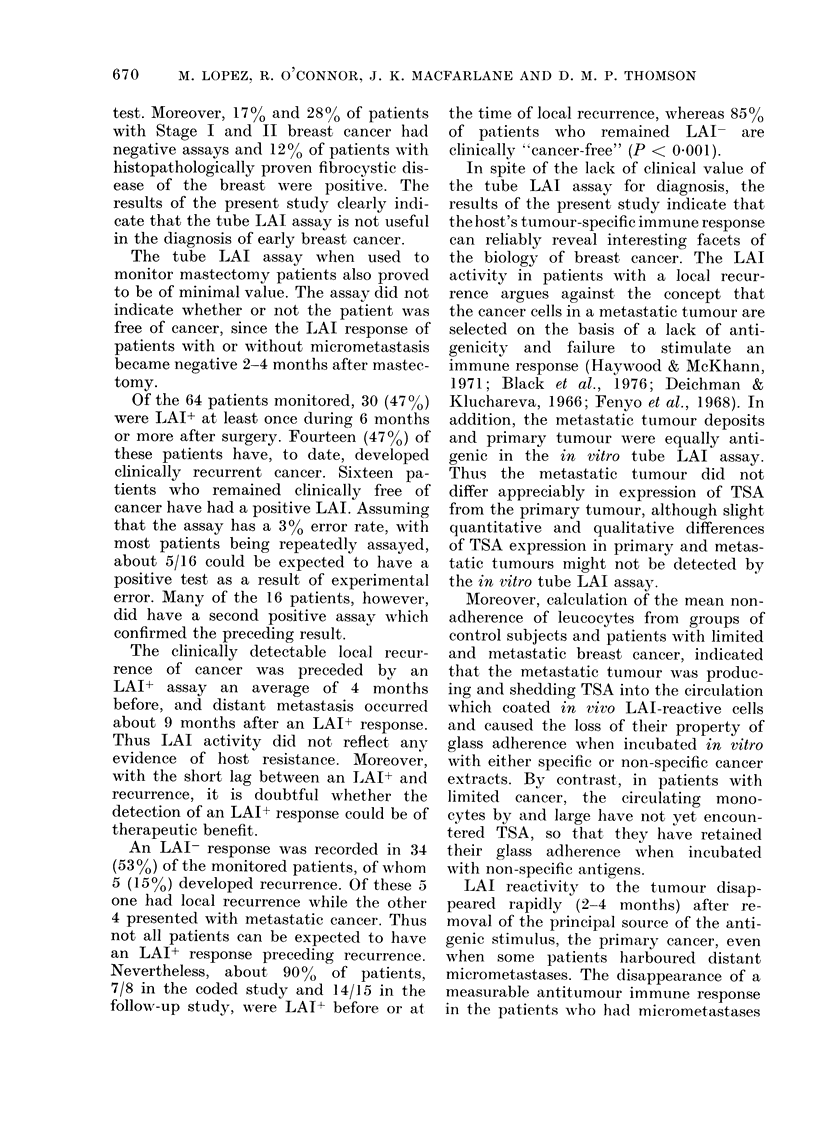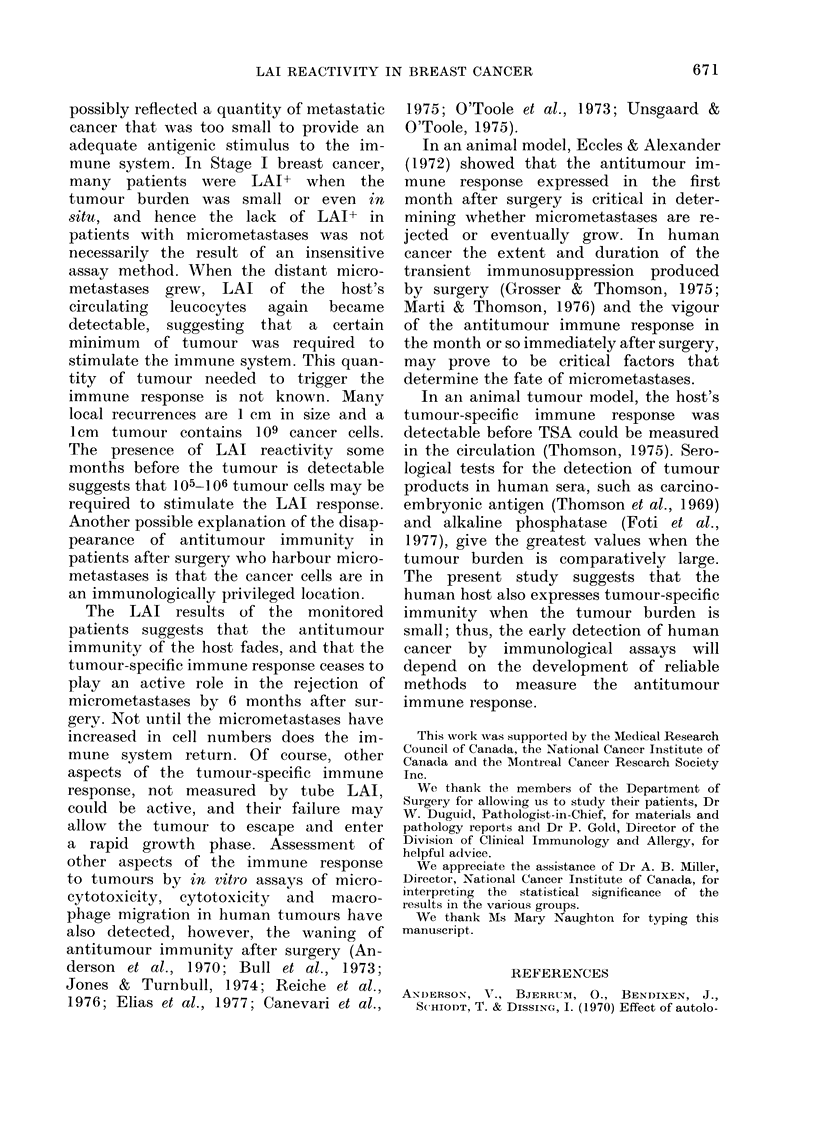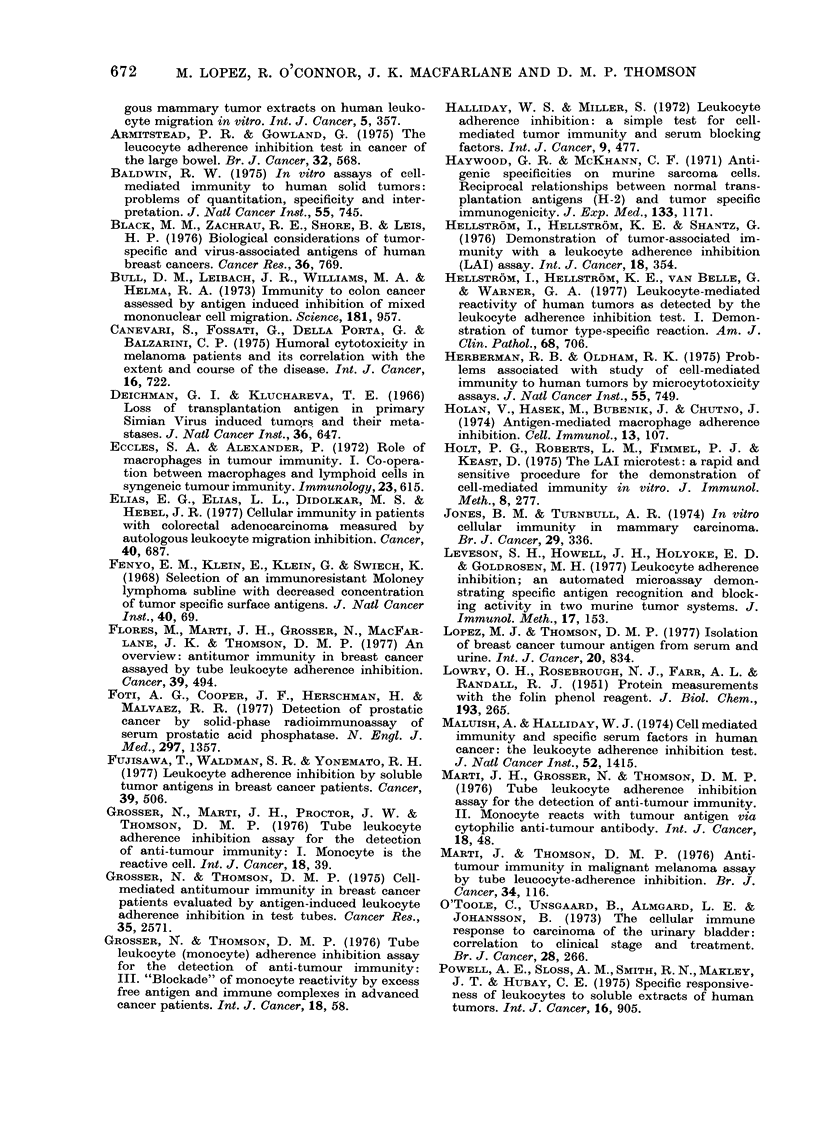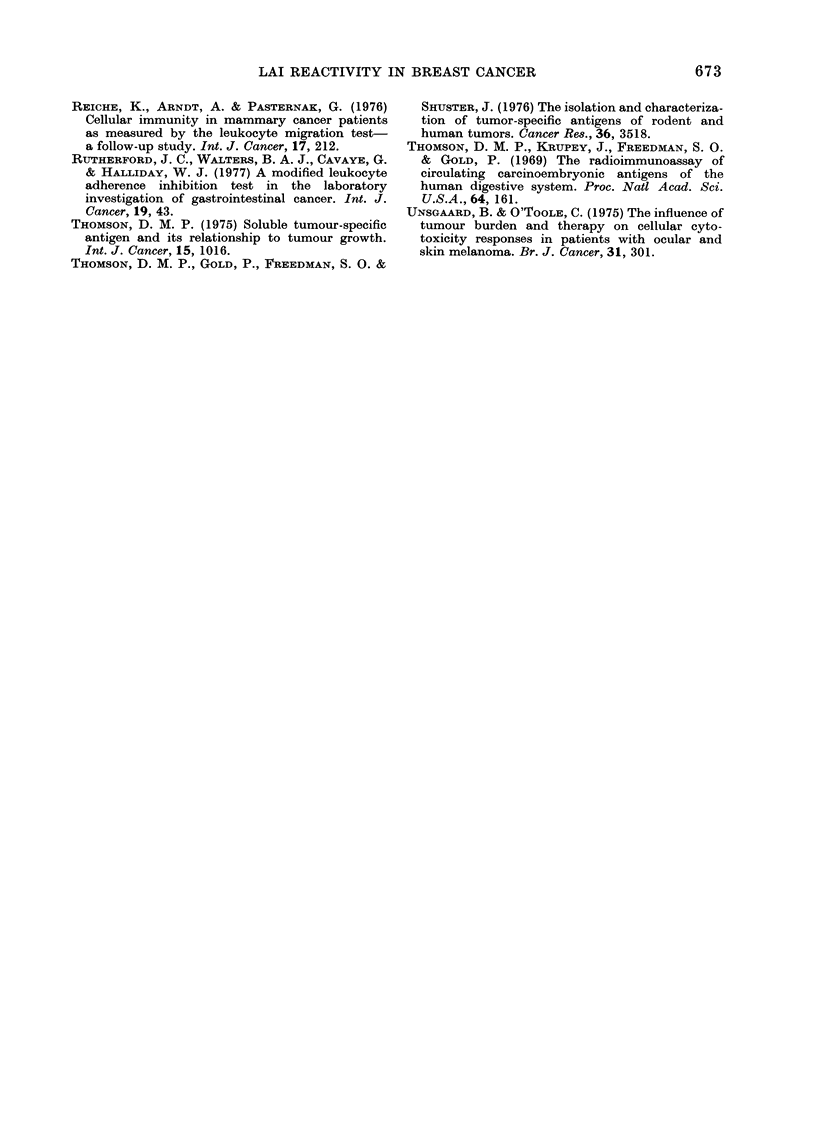# The natural history of antitumour immunity in human breast cancer assayed by tube leucocyte adherence inhibition.

**DOI:** 10.1038/bjc.1978.271

**Published:** 1978-12

**Authors:** M. Lopez, R. O'Connor, J. K. MacFarlane, D. M. Thomson

## Abstract

The specificity of the tube LAI in breast cancer was examined in a study with coded samples of PBL. In addition, 64 patients with breast cancer had their LAI reactivity monitored and correlated with their clinical status for up to 3 years after mastectomy. When patients were assayed by tube LAI, 83, 72, and 29% with Stage I, and II and III breast cancer respectively were positive. In Stage IV brest cancer, 88% of those with local recurrence and 15% of those with disseminated cancer were positive. By contrast, 3% of control subjects were LAI+. A select group of patients admitted to hospital with suspicious breast lumps that histopathologically proved to be benign breast disease (BBD) had a higher incidence of LAI+ (12%), whereas of outpatients with BBD only 2% were LAI+. Most breast cancer patients LAI reactivity became negative 2--4 months after mastectomy, even when some harboured micrometastases. LAI reactivity remained absent in those patients who remained clinically "cancer-free". In the follow-up patients, LAI activity returned about 4 months before local recurrence. LAI reactivity was observed in 7/8 patients in the coded study and 14/15 patients in the follow-up study preceding and/or at the time of local recurrence. A few patients (15%) progressed to widespread cancer without preceding positive LAI activity. The results suggest that tumour-specific immunity rapidly fades after surgery and may play no role in the rejection of micrometastases by 6 months after surgery. In addition, the present study has shown that the human hose manifests tumour-specific immunity when the cancer is small, and suggests that the early detection of human cancer would depend upon reliable methods to measure the tumour-specific immune response.


*Br. J. Cancer* (1978) **38**, 660

### THE NATURAL HISTORY OF ANTITUMOUR IMMUNITY IN HUMAN BREAST CANCER ASSAYED BY TUBE LEUCOCYTE ADHERENCE INHIBITION

M. LOPEZ, R. O'CONNOR, J. K. MACFARLANE AND D. M. P. THOMSON*

(With Technical Assistance of P. Friedlander and J. Weatherhead)

*From the Montreal General Hospital Research Institute, Division of Clinical Immunology and Department of Surgery, Montreal General Hospital, McGill University, Montreal, Canada H3G 1A4*

Received 5 June 1978 Accepted 4 September 1978

**Summary.—The specificity of the tube LAI in breast cancer was examined in a study with coded samples of PBL. In addition, 64 patients with breast cancer had their LAI reactivity monitored and correlated with their clinical status for up to 3 years after mastectomy. When patients were assayed by tube LAI, 83, 72, and 29% with Stage I, and II and III breast cancer respectively were positive. In Stage IV breast cancer, 88% of those with local recurrence and 15% of those with disseminated cancer were positive. By contrast, 3% of control subjects were LAI+. A select group of patients admitted to hospital with suspicious breast lumps that histopathologically proved to be benign breast disease (BBD) had a higher incidence of LAI+ (12%), whereas of outpatients with BBD only 2% were LAI+. Most breast cancer patients' LAI reactivity became negative 2–4 months after mastectomy, even when some harboured micrometastases. LAI reactivity remained absent in those patients who remained clinically "cancer-free". In the follow-up patients, LAI activity returned about 4 months before local recurrence. LAI reactivity was observed in 7/8 patients in the coded study and 14/15 patients in the follow-up study preceding and/or at the time of local recurrence. A few patients (15%) progressed to widespread cancer without preceding positive LAI activity. The results suggest that tumour-specific immunity rapidly fades after surgery and may play no role in the rejection of micrometastases by 6 months after surgery. In addition, the present study has shown that the human host manifests tumour-specific immunity when the cancer is small, and suggests that the early detection of human cancer would depend upon reliable methods to measure the tumour-specific immune response.**

DURING the past 4 years our laboratory has assessed specific immunity to human breast cancer by the *in vitro* assay of tube leucocyte adherence inhibition (tube LAI) (Grosser & Thomson, 1975, 1976; Marti & Thomson, 1976; Grosser *et al.*, 1976; Marti *et al.*, 1976; Flores *et al.*, 1977; Thomson *et al.*, 1976). This assay was modified from that of Holan *et al.* (1974) and is based on the discovery of the leucocyte adherence inhibition (LAI) phenomenon by Halliday & Miller (1972). Many other investigators who have used LAI to study the host's immune response to animal and human tumours have reported that the assay reliably measures the tumour-specific immune response of the host (Holt *et al.*, 1975; Leveson *et al.*, 1977; Maluish & Halliday, 1974; Powell *et al.*, 1975; Fujisawa *et al.*, 1977; Rutherford *et al.*, 1977). In contrast, Hellström *et al.* (1977), who used the tube LAI assay in malignant melanoma, found that although the response was tumour-specific, there was too much non-specificity to be of clinical value. Also, stage of

* To whom reprint requests should be addressed: Dr D. M. P. Thomson, Montreal General Hospital, 1650 Cedar Avenue, Montreal, Canada H3G 1A4.

disease, amount of clinically detectable tumour and clinical status did not influence their LAI results. Similarly, Hellström *et al.* (1976), in an experimental animal-tumour model, found specific LAI reactivity, but great variation between individual tests. Likewise, Armitstead & Gowland (1975) also reported lack of specificity of an LAI assay in colon cancer, where 59% of colonic-cancer patients and 30% of control subjects showed LAI to a perchloric-acid extract of colonic cancer.

Our studies indicated that in cancer patients, tumour-specific immunity was generally present with small tumour burdens, and was more often absent from widespread metastasis (Grosser & Thomson, 1975, 1976; Marti & Thomson, 1976; Marti *et al.*, 1976; Thomson *et al.*, 1976). In patients with limited cancer, removal of the primary tumour was followed within 3–4 months by a loss of LAI reactivity in patients considered to be clinically "cancer-free" (Marti & Thomson, 1976; Flores *et al.*, 1977). Leucocytes of patients with advanced cancer were not LAI$^+$ because free breast-tumour-specific antigen (TSA) in the systemic circulation coated the cell surface of the leucocyte and abrogated the response to antigen present in the *in vitro* assay (Grosser & Thomson, 1976; Lopez & Thomson, 1977). Moreover, the breast TSA shed from the tumour-cell surface was present in serum and co-isolated with the high-density lipoproteins and HLA antigens (Lopez & Thomson, 1977). In addition, some of the excess TSA in the systemic circulation was degraded and cleared by glomerular filtration into the urine (Lopez & Thomson, 1977).

In the present study, the relationship of tumour immunity to clinical behaviour of the cancer up to 3 years after mastectomy was examined in 64 patients, in an attempt to increase the understanding of the biological events of tumour growth and antitumour immunity that follow excision of the primary cancer. The present study in breast cancer and control subjects was also undertaken to determine the specificity of the tube LAI assay when leucocyte samples were coded.

### MATERIALS AND METHODS

*Subjects.*—The TNM classification by the International Union Against Cancer for clinicopathological staging of breast cancer was used. Patients admitted to hospital with breast masses, patients with a mastectomy admitted for investigation or treatment of local recurrence of breast cancer or disseminated disease, as well as various control groups, were tested for tube LAI before surgery or any other treatment. A total of 451 patients were assayed, of whom 139 had new breast lesions, benign or malignant. A further group of 41 outpatients suspected of breast pathology, who were referred for mammography, were assayed for tube LAI. Control subjects who were tested were divided into 2 groups: 92 patients with malignant tumours other than breast cancer, and 138 patients with benign surgical disorders.

In the second part of the study, 64 patients who underwent mastectomy were monitored by the tube LAI every 3–6 months, with some patients being monitored for over 3 years. The patients studied were those who were LAI$^+$ before surgery and who returned for LAI blood tests. The pathological stages of these patients at surgery was 27 Stage I, 31 Stage II and 6 Stage III. Most of the LAI values were correlated with the clinical results at the end of the first, second and third year of the study. However, when a patient was LAI$^+$ this was reported to the attending surgeon and the LAI result was then correlated with the clinical status of the patient. In this study, a single NAI value of $\geqslant 30$ 6 months or more after surgery resulted in the patient being recorded as a positive responder. The patients were examined by their private physician or at the Oncology Clinic of the Montreal General Hospital every 3 months.

*Tumour extracts.*—Phosphate-buffered saline (PBS, pH 7·3) extracts of malignant melanoma and cancer of the breast, colon, stomach and pancreas were prepared as described by Grosser & Thomson (1975). The protein concentrations were determined by the method of Lowry *et al.* (1951) with bovine albumin as a standard, and the extracts of the specific cancer (breast) and the controls (melanoma) were titrated against peripheral blood leucocytes (PBL) from reactive breast-cancer patients and control subjects as previously described (Marti & Thomson, 1976; Flores *et al.*, 1977). The optimal protein concentration was about $100 \pm 10\,\mu g$ per tube. In the coded study conducted over a period of 1 year, the breast-cancer extract was made from portions of the same breast cancer. During the 3-year follow-up study, a variety of cancer extracts, specific and non-specific, were prepared and used for the study. Most extracts prepared from breast cancers and malignant melanoma have had activity when used in the tube LAI assay, though some extracts have seemed better than others in terms of consistent non-specific inhibition of adherence of leucocytes from control subjects. There was no discernible difference in the preparations selected for use in this study. Except for the study on the tumour specificity of the LAI response, routine testing was performed with the extracts of breast cancer and malignant melanoma.

*The antigen-induced tube leucocyte-adherence-inhibition assay* (*tube LAI assay*).—The tube LAI assay was performed as described in detail by Grosser & Thomson (1975). PBL from breast-cancer patients and control subjects were collected and prepared as previously described (Grosser & Thomson, 1975). The cells were then plated in separate glass test tubes with the specific and control antigens, and incubated in a horizontal position at 37°C in a 5% $CO_2$ humidified atmosphere. After 2 h the tubes were placed vertically, and a sample of the non-adherent cells were counted on a haemocytometer with a Leitz Dialux microscope with phase contrast.

The results were expressed as a nonadherence index (NAI)

$$\mathrm{NAI} = \frac{\begin{array}{l}\text{Non-adherent cells in}\\ \text{presence of specific}\\ \text{antigen}\end{array} - \begin{array}{l}\text{Non-adherent cells in}\\ \text{presence of non-specific}\\ \text{antigen}\end{array}}{\text{Non-adherent cells in presence of non-specific antigen}} \times 100$$

An NAI value $\geqslant 30$ was considered positive, on the basis that more than 95% of the control population was negative, and more than 80% of patients with limited cancer were positive to the sensitizing antigen (Marti & Thomson, 1976; Flores *et al.*, 1977). PBL samples were coded by laboratory personnel not involved in the clinical studies of LAI. The tests were performed before surgery, and all patients with breast lumps were tested together with one or more control subjects. The LAI results for each patient were known on the day of the LAI test, and were correlated with the pathological results which became available 7–10 days later. The tube LAI assay was performed independently by M. Lopez and R. O'Connor and the results of the 2 investigators were pooled.

### RESULTS

### *LAI response directed to an organ-type-specific antigen*

Routinely, a pair of extracts was appropriately titrated (Marti & Thomson, 1976) for use in the tube LAI assay. The pairs of cancer extracts consisted of breast and melanoma and, more recently, colon and lung, pancreas and lung, and stomach and lung. The extract of malignant melanoma was the non-specific antigen in the coded study of LAI reactivity to breast cancer. Nevertheless, the extract of malignant melanoma showed specific antigen activity when incubated with leucocytes from patients with malignant melanoma. By studying the tumour-specific immune response to one pair of extracts, it has proved possible to maintain the experimental error (false positives) to about 3% (O'Connor *et al.* in press) to one antigen of the pair. The different cancer extracts can be freely interchanged; if, however, the number of extracts to which a patient is tested is increased, the experimental error may be expected to rise as a result of more assays being done. In addition, the percentage of patients positive to a single antigen of a panel of antigens will also rise. Table I shows the results when leucocytes from patients with limited cancer and control subjects

TABLE I.—*LAI reactivity to a panel of cancer extracts of different histological origins**

| Diagnosis of leucocyte donor | Mean No. ± s.d. of non-adherent cells to a panel of cancer extracts of | | | | | |
|---|---|---|---|---|---|---|
| | Breast | Melanoma | Colon | Pancreas | Stomach | Lung |
| 1. Breast cancer | 79±11 | 58±5 | 59±9 | 55±1 | 50±2 | 48±5 |
| 2. Malignant melanoma | 43±2 | 67±5 | 48±6 | 41±3 | 49±5 | 50±5 |
| 3. Colon cancer | 28±3 | 26±4 | 39±3 | 28±2 | 30±2 | 28±1 |
| 4. Pancreatic cancer | 30±3 | 27±5 | 29±7 | 49±8 | 26±3 | 23±4 |
| 5. Lung cancer | 40±9 | 34±5 | 38±7 | 46±9 | 44±6 | 78±13 |
| 6. Control subject | 23±4 | 28±1 | 30±4 | 30±3 | 26±4 | 25±2 |
| 7. Control subject | 51±2 | 57±14 | 49±8 | 54±11 | 50±5 | 52±2 |
| 8. Control subject | 41±6 | 41±3 | 43±13 | 45±5 | 43±6 | 44±4 |
| 9. Control subject | 50±12 | 54±1 | 67±14 | — | — | 59±9 |
| 10. Control subject | 62±11 | 49±7 | 53±6 | 51±3 | 64±11 | 59±15 |

* NAI is calculated as $\frac{A-B}{B} \times 100$, where A = specific antigen and B = non-specific antigen. Hence, LAI reactivity to each antigen can be calculated 5 ways with the other 5 antigens.

were incubated with 6 different cancer extracts. The highest number of donor leucocytes were non-adherent when incubated with an extract of cancer identical to the tumour of the donor. Moreover, in the patients with cancer an NAI value $\geqslant 30$ was observed when the specific antigen was paired with any of the non-specific antigens. Each patient with cancer had, therefore, a total of 5 positive tests to the sensitizing cancer antigen.

If the various antigens are freely interchanged to calculate the NAI, the results show 6 false-positive responses (Table I). NAI values between 30 and 35 are given to breast-cancer antigen by the *Breast/Lung* combination, in Patient No. 4 with pancreatic cancer; to pancreas-cancer antigen by the *Pancreas/Melanoma* combination in Patient No. 5 with lung cancer; to colon-cancer antigen and pancreas-cancer antigen by the combination of *Colon/Breast*, *Pancreas/Breast*, respectively in Control Subject No. 6; to colon-cancer antigen by the *Colon/Breast* combination in Control Subject No. 9; to stomach-cancer antigen by the combination of *Stomach/Melanoma* in Control Subject No. 10. By our method of pairing the tumour extracts to calculate the NAI, if each patient was tested with 6 extracts, this resulted in a total of 30 permutations of tests for each patient, and the 10 patients had 262 tests. The 6 false-positive results represented, therefore, an experimental error of 2·1%. Although the experimental error was low, 3/6 control subjects had an LAI+ response to one of the antigens and 2/4 patients with cancer reacted falsely to an unrelated extract of cancer. It was of interest that the false positives occurred not within the standard pairs but between the pairs, in part, perhaps because the extracts of each of the pairs had not been as carefully titrated against the other pairs.

In addition, Table II shows that 92 patients with a variety of limited cancers were tested against the pair of extracts of breast cancer and melanoma with 3 positive responses (3%) to the breast-cancer extract. In spite of the false-positives recorded in Table I, the results

TABLE II.—*Coded study of tube LAI assay in breast-cancer patients and control subjects**

| Diagnosis of patients studied | No. tested | LAI+ patients No. (%) |
|---|---|---|
| Breast cancer | | |
| Stage I | 24 | 20 (83)† |
| Stage II | 25 | 18 (72)† |
| Stage III | 14 | 4 (29) |
| Stage IV | | |
| Local recurrence | 8 | 7 (88) |
| Disseminated | 33 | 5 (15)‡ |
| Benign breast disease | | |
| In-hospital patients | 76 | 9 (12) |
| Outpatients | 41 | 1 (2) |
| Unrelated malignancies | 92 | 3 (3) |
| Non-malignant surgical diseases | 138 | 4 (3) |

* Patients with Stage I, II, III breast cancer, with unrelated malignancies, with non-malignant surgical diseases, and with benign breast disease (in-hospital) were tested before surgery. Stage IV patients were tested before surgery, irradiation or chemotherapy.
† Significantly different from control subjects ($P < 0{\cdot}001$).
‡ Significantly different from Stage I and II breast cancer patients ($P < 0{\cdot}01$).

indicate that the LAI response is directed to an organ-specific antigen.

Another feature of the tube LAI assay was that the PBL from different donors exhibited variable non-adherence (Table I) which also varied each day. To detect a tumour-specific immune response it was essential, therefore, to compare the difference in non-adherence when the PBL were incubated with specific and non-specific extracts of cancer.

*A coded study of tube LAI*

A total of 451 patients were tested independently by 2 experimenters (Table II). The LAI results of the 2 experimenters were similar for both control subjects and patients with breast cancer, so it has been possible to pool the results (summarized in Table II). In addition, many of the same patients with breast cancer were tested by both investigators either on the same or separate days with similar results. The similar results by both investigators when testing the same patient or when their overall results were compared indicated that the tube LAI assay was reproducible.

Of the group of patients tested, 139 were admitted to hospital with a breast mass which was thought to be malignant by the attending surgeon. The pathology of the excised breast tissue showed that 24, 25 and 14 had Stage I, II and III breast cancer, respectively, though 76 proved to have benign breast lesions. Before surgery, 83% of Stage I, 72% of Stage II, and 29% of Stage III patients were LAI+ (Table II). By contrast, 12% of the patients with benign breast lesions had a positive LAI test before surgery.

A positive LAI response was exhibited by 4 patients who had no palpable breast mass, but were admitted to hospital because of mammographic suspicion of breast cancer. At surgery they were found to have breast cancer. In addition one patient included in Stage I who had lobular *in-situ* breast cancer was LAI+.

Patients with Stage IV breast cancer were divided by clinical evaluation into patients with local recurrence and widespread metastasis. Of those with local recurrence, tested before the recurrence was proven by biopsy, 7/8 (88%) were LAI+, whereas only 5/33 (15%) patients with widespread metastasis were LAI+ (Table II).

The rate of LAI+ for the group of patients with breast masses who proved to have benign breast disease (BBD) was higher than in the other control subjects. To determine whether all BBD patients showed a similar high degree of LAI

reactivity to the breast-cancer extract, a second group of 41 patients attending a mammography outpatient clinic were tested. The clinical and mammographic diagnosis in these 41 patients was later correlated with the LAI results, and all the patients had BBD. Of these 41, one (2%) was LAI$^+$ (Table II), but, when this patient was assayed on 2 subsequent occasions, she was LAI$^-$, and the response to a panel of breast cancer antigens was also negative (O'Connor *et al.*, in press).

In the control groups, 92 patients who had malignant lesions other than of the breast were assayed (Table II). This group comprised a variety of gastrointestinal cancers, including pancreas, colon, stomach and hepatomas, as well as squamous-cell carcinomas of the floor of the mouth, larynx and bronchi, melanoma, ovarian cancers, sarcomas and lymphomas. All patients were tested before surgery or treatment. Three patients in this group were LAI$^+$ (Table II). One was a woman originally admitted to hospital for a breast lesion that was histologically benign on biopsy, and while in hospital was found to have ovarian cancer which had metastasized to omentum. She was included as a false-positive in the unrelated-malignancy group. The second false-positive was a man with lung cancer who had a positive response to breast-cancer extract because of an abnormally low cell count with the melanoma antigen, the non-specific antigen. However, when leucocytes of this patient were tested against extracts of lung cancer and breast cancer, an LAI$^+$ response to lung cancer and not to breast cancer was observed. The third LAI$^+$ patient had a negative response when retested twice more, suggesting that the initial result was an experimental error.

Patients in the second group of control subjects, with a variety of benign surgical conditions, were tested before surgery and 4/138 (3 women and 1 man) were LAI$^+$. No relationship was found with either family history of breast cancer or symptoms of breast disease. Moreover, 3/4 positive responders, when retested on 2 more occasions, were negative, suggesting that the initial LAI$^+$ was an experimental error (O'Connor *et al.*, in press). The combined false-positive rate in the 2 groups of control subjects was 3%. Hence, the NAI values are less than 30 in more than 95% of instances, when leucocytes of control subjects are tested against breast cancer as the specific antigen. In addition, about 3% of control subjects without malignant melanoma had NAI values less than — 30 and would be considered false-

TABLE III.—*Mean number of non-adherent leucocytes from control subjects and patients with limited and metastatic breast cancer to extracts of breast and melanoma cancer*

| Groups‖ | Non-adherent cells to breast cancer | | | Non-adherent cells to melanoma | | | |
|---|---|---|---|---|---|---|---|
| | Mean ± s.d.* | Coeff. of variation† | P‡ | Mean ± s.d.* | Coeff. of variation† | P‡ | NAI§ |
| 1. Control subjects | 37 ± 12 | 32 | 1 *vs* 3 <0·05 | 37 ± 12 | 32 | 1 *vs* 3 <0·02 | 0 |
| 2. Patients with early breast cancer | 60 ± 10 | 16 | 2 *vs* 1 <0·001 | 37 ± 8 | 21 | 2 *vs* 1 N.S. | 59 |
| 3. Patients with metastatic breast cancer | 50 ± 27 | 54 | 3 *vs* 2 <0·05 | 51 ± 28 | 55 | 3 *vs* 2 <0·02 | −3 |

* The mean of non-adherent samples of cells from the 30 test tubes in the 10 patients.
† s.d./Mean %.
‡ Student's *t* test between groups.
§ Calculated from the means.
‖ Ten patients in each group.

positive to the non-specific antigen of malignant melanoma.

*Mean antigen-induced non-adherence of leucocytes from breast-cancer patients*

Breast cancer patients and control subjects tested during a period of about 3 months with the same specific and non-specific tumour extracts were allocated to 3 groups (Table III). Group 1 was control subjects with benign surgical conditions. Group 2 was $LAI^+$ patients with localized breast cancer. Group 3 was patients with metastatic breast cancer and $LAI^-$ (Table III). In this analysis, the breast-cancer patients were chosen consecutively until each group had 10 patients. The group of control subjects consisted of those who were tested on the same day as the selected breast-cancer patients.

Table III shows the mean leucocyte non-adherence of the 3 groups. Leucocytes from Group 1 were equally non-adherent when incubated with the 2 cancer extracts. PBL from Group 2 incubated with the non-specific cancer extract had a mean non-adherence similar to Group 1. By contrast, the leucocytes from Group 2 incubated with an extract of breast cancer, the sensitizing cancer, had a mean non-adherence significantly higher ($P < 0{\cdot}001$).

Leucocytes from Group 3 showed a mean non-adherence significantly higher than Group 1, when incubated with the extracts of breast cancer and melanoma (Table III). The mean non-adherence of leucocytes from Group 3 patients was lower, however, than in Group 2 when incubated with the extract of breast cancer. When more groups of patients were similarly examined, the results were the same, except that the mean non-adherence of leucocytes from Group 3 patients to the specific and non-specific antigens were almost equal to the mean non-adherence of leucocytes from Group 2 patients when incubated with the specific antigen.

*Follow-up of patients after mastectomy by the tube LAI assay*

Included in this study were 64 patients who were $LAI^+$ before surgery (Table IV) and in 62 of these the LAI became negative 2–4 months after surgery. The patients were divided according to the month after surgery when the study was closed (Table IV). To date, 30 patients (47%) subsequently had a positive LAI

TABLE IV.—*Follow-up of 64 mastectomy patients by the tube LAI: correlation between LAI response and cancer recurrence*

| Time after mastectomy (months) | No. tested | $LAI^+$ patients | | | $LAI^-$ patients | | |
|---|---|---|---|---|---|---|---|
| | | Total (%) | Loc. rec.* | Prog. diss.† | Total (%) | Loc. rec.* | Diss. Ca.‡ |
| 12–18 | 7 | 6 (86) | 4 | 3 | 1 (14) | 0 | 1§ |
| 18–24 | 20 | 10 (50) | 5 | 4 | 10 (50) | 1 | 1‖ |
| 24–36 | 26 | 10 (38) | 2 | 2 | 16 (61) | 0 | 0 |
| 36+ | 11 | 4 (36) | 3 | 2 | 7 (64) | 0 | 2¶ |
| Total | 64 | 30 (47) | 14** | 11†† | 34 (53) | 1** | 4†† |

* Patients with local recurrence.
† Patients who progressed to dissemination.
‡ Patients with disseminated cancer who never had evidence of local recurrence.
§ Patient without documented local recurrence, but presented with widespread cancer. $LAI^-$ throughout.
‖ Patient with Stage I cancer at surgery, presented 1 year after surgery with a large ovarian mass which proved metastatic from breast. $LAI^-$ throughout.
¶ Two patients lost to follow-up for one year. Both appeared for clinical examination showing disseminated disease. $LAI^-$ at the clinical investigation.
** The difference between the proportions of patients with recurrent disease who were $LAI^+$ and $LAI^-$ is highly significant. $\chi^2$ 14·63; $P < 0{\cdot}001$).
†† The difference between the proportions who progressed to dissemination between $LAI^+$ and $LAI^-$ patients is less significant ($\chi^2$ 4·21; $P < 0{\cdot}05$).

6 months or more after surgery. Of these, 14 have had histologically proven local recurrence of breast cancer and 11 of these 14 have eventually progressed to widely disseminated breast cancer. The remaining 16 LAI+ patients have been followed for an average of 4 months, and as yet have no clinically detectable recurrence.

In the group of 34 (53%) patients who have remained LAI− 6 months or more after surgery, 5 (15%) presented with clinical evidence of local or disseminated cancer (Table IV). One patient had clinically evident ovarian metastasis 11 months after surgery and another showed metastatic disease 20 months after surgery, both having repeatedly LAI− assays throughout the monitoring period. The third and fourth patients were lost to LAI follow-up for 1 year, after which they presented with metastatic cancer. No local recurrence of breast cancer was demonstrated in these 4 patients with a negative LAI. The 1 LAI− patient with local recurrence was tested at 5 months, on the day before biopsy was carried out on the local recurrence. Many of the remaining patients in this LAI− group presumably have no residual cancer, though the possibility that some harbour undetectable micrometastasis cannot be excluded.

Table V shows the results when the 64 follow-up breast-cancer patients were grouped according to the stage of cancer at surgery, their LAI response 6 months or more after mastectomy and the recurrence of cancer. The percentage of patients who became LAI+ 6 months or more after surgery was highest in Stage III cancer and lowest in Stage I (Table V). Likewise, the development of proven local recurrence in the LAI+ patients was highest in Stage III and lowest in Stage I (Table V). The highest percentage of LAI+ patients with no evidence of recurrent cancer was found in the patients with Stage I breast cancer. Only 20% of Stage I LAI+ patients have to date developed local recurrence, whereas 75% of LAI+ Stage III have done so.

In the patients who have developed recurrences, the LAI response became positive about 4 months (range 1–7) before the local recurrence was detected. In addition, the LAI+ patients who developed local recurrence had a negative LAI value about 4 months (range 1–13) before the positive test. In the group of LAI+ patients with local recurrence, LAI reactivity was recorded about 9 months (range 4–13) before the appearance of distant metastasis.

*Patterns of LAI reactivity in follow-up mastectomy patients*

Figs. 1–5 show the 5 patterns of LAI reactivity observed when patients were monitored before and after mastectomy. Fig. 1 illustrates patients progressing rapidly to the metastatic stage of cancer. Two patients were LAI+ at 6 or 7 months, and shortly after presented with clinical evidence of recurrent cancer, though as the tumour grew LAI reactivity was lost and clinical evidence of disseminated cancer followed. The third patient was LAI+ before surgery but later showed no LAI reactivity and presented within a year with evidence of disseminated breast

TABLE V.—*Stage at initial surgery, LAI response* 6 *months or more after mastectomy and cancer recurrence in* 64 *follow-up breast-cancer patients*

| Stage of cancer | No. tested | LAI+ patients | | | LAI− patients | | |
|---|---|---|---|---|---|---|---|
| | | Total (%) | Loc. rec.* | Prog. diss.† | Total (%) | Loc. rec.* | Diss. ca.‡ |
| I | 27 | 10 (37) | 2 | 1 | 17 (63) | 0 | 1 |
| II | 31 | 16 (52) | 9 | 7 | 15 (48) | 1 | 3 |
| III | 6 | 4 (67) | 3 | 3 | 2 (33) | 0 | 0 |

* Patients with local recurrence.
† Patients who progressed to dissemination.
‡ Patients with disseminated cancer who never had evidence of local recurrence.

Fig. 1.—LAI of leucocytes from 3 patients with local recurrence within 6–12 months of surgery.

Fig. 3.—LAI becoming positive more than 6 months after surgery in patients who are clinically "cancer-free".

Fig. 2.—LAI in 4 patients who are clinically "cancer-free".

Fig. 4.—LAI becoming positive more than 6 months after surgery in patients who later presented with local recurrence.

cancer. Fig. 2 illustrates patients who to date have not developed clinically detectable recurrence of breast cancer. Invariably, these patients become LAI$^-$ 2–4 months after surgery and remain negative. This does not differ from the pattern of the third patient in Fig. 1, except that the patients in Fig. 2 have remained clinically "cancer-free". Figs. 3 and 4 illustrate the third and fourth patterns. Here, although the patients are

Fig. 5.—LAI becoming positive with local recurrence and then negative as the tumour burden grows.

LAI$^-$ in the 2–4 months after surgery, they become LAI$^+$ again after 6 months. Fig. 3 shows 3 LAI$^+$ patients who are clinically "cancer-free". In Fig. 4 a similar pattern is seen, except that local recurrence of breast cancer has now become evident. Fig. 5 shows the sequence of events in patients who develop local recurrence more than 1 year after mastectomy. The LAI$^+$ response before surgery becomes negative 2–4 months after surgery and remains negative until about 4–5 months before the local recurrence is detected clinically. The LAI response remains positive for a variable time, but the change from LAI$^+$ to LAI$^-$ usually precedes evidence for clinical dissemination.

### DISCUSSION

The validity of the *in vitro* microcytotoxicity assay for assessing whether human tumours do indeed express neoantigens capable of host recognition has recently been questioned (Baldwin, 1975; Herberman & Oldham, 1975). Partly as a response to the difficulties experienced with the various methods for measuring *in vitro* cytotoxicity of PBL from cancer patients, attention has been directed towards developing alternative *in vitro* tests of cellular immunity to human tumours. The phenomenon of tumour-antigen-induced inhibition of leucocyte adherence to glass (Halliday & Miller, 1972) appears to be a most promising *in vitro* technique. The results of the present study indicate that the assay is able to detect a host response to an organ-specific neoantigen expressed upon human cancers.

The results of the present study of tube LAI in breast-cancer patients and control subjects, in whom the PBL samples were coded and tested independently by 2 investigators, are similar to previous results from our laboratory (Grosser & Thomson, 1975; Flores *et al.*, 1977). A high percentage of patients with small tumour burdens or early cancer were LAI$^+$, 83% in Stage I and 72% in Stage II, compared with only 15% of patients with large tumour burdens, Stage IV, with widespread metastasis. In this and our previous study (Flores *et al.*, 1977) patients with Stage III cancer had decreased reactivity, 29 and 45%, respectively. On the other hand, about 88% of patients with Stage IV cancer with local recurrence, compared to 15% with widespread metastasis, were LAI$^+$. In the patients with local recurrence, the tumour burden was apparently not large enough to release sufficient TSA to abrogate the LAI response (Grosser & Thomson, 1976; Lopez & Thomson, 1977). In Stage IV cancer, the presence of LAI reactivity suggested a limited tumour load, whereas absence of a LAI response signified a large tumour load.

About 3% of control subjects had a positive LAI to the breast-cancer extract. Most of the control subjects with a false LAI$^+$ were shown to be the result of experimental error. Thus, the experimental error in the tube LAI assay to a single antigen of the pair of extracts is about 3%. If the number of extracts to which the patients are tested is increased, the rate of false positives may rise. With 6 different cancer antigens and the use of our formula, there are 30 permutations by which the NAI can be calculated for the non-adherent PBL from one subject incubated with the 6 antigens. An experimental error rate of 2·5% will lead to more than 50% of tested control subjects being apparently positive once in the 30 possible permutations of the 6 antigens. By contrast, PBL from a tumour-bearing patient with tumour-specific immunity can be expected to be positive to the sensitizing antigen in 5/5 possible combinations in which the sensitizing antigen is the specific antigen, out of the 30 different permutations for calculating the NAI, a result which is markedly different from the control subjects. Nevertheless, in the present state of development of the tube LAI, the use of a panel of cancer extracts has little applicability as a cancer-screening test. Moreover, 17% and 28% of patients with Stage I and II breast cancer had negative assays and 12% of patients with histopathologically proven fibrocystic disease of the breast were positive. The results of the present study clearly indicate that the tube LAI assay is not useful in the diagnosis of early breast cancer.

The tube LAI assay when used to monitor mastectomy patients also proved to be of minimal value. The assay did not indicate whether or not the patient was free of cancer, since the LAI response of patients with or without micrometastasis became negative 2–4 months after mastectomy.

Of the 64 patients monitored, 30 (47%) were $LAI^+$ at least once during 6 months or more after surgery. Fourteen (47%) of these patients have, to date, developed clinically recurrent cancer. Sixteen patients who remained clinically free of cancer have had a positive LAI. Assuming that the assay has a 3% error rate, with most patients being repeatedly assayed, about 5/16 could be expected to have a positive test as a result of experimental error. Many of the 16 patients, however, did have a second positive assay which confirmed the preceding result.

The clinically detectable local recurrence of cancer was preceded by an $LAI^+$ assay an average of 4 months before, and distant metastasis occurred about 9 months after an $LAI^+$ response. Thus LAI activity did not reflect any evidence of host resistance. Moreover, with the short lag between an $LAI^+$ and recurrence, it is doubtful whether the detection of an $LAI^+$ response could be of therapeutic benefit.

An $LAI^-$ response was recorded in 34 (53%) of the monitored patients, of whom 5 (15%) developed recurrence. Of these 5 one had local recurrence while the other 4 presented with metastatic cancer. Thus not all patients can be expected to have an $LAI^+$ response preceding recurrence. Nevertheless, about 90% of patients, 7/8 in the coded study and 14/15 in the follow-up study, were $LAI^+$ before or at the time of local recurrence, whereas 85% of patients who remained $LAI^-$ are clinically "cancer-free" ($P < 0{\cdot}001$).

In spite of the lack of clinical value of the tube LAI assay for diagnosis, the results of the present study indicate that the host's tumour-specific immune response can reliably reveal interesting facets of the biology of breast cancer. The LAI activity in patients with a local recurrence argues against the concept that the cancer cells in a metastatic tumour are selected on the basis of a lack of antigenicity and failure to stimulate an immune response (Haywood & McKhann, 1971; Black *et al.*, 1976; Deichman & Kluchareva, 1966; Fenyo *et al.*, 1968). In addition, the metastatic tumour deposits and primary tumour were equally antigenic in the *in vitro* tube LAI assay. Thus the metastatic tumour did not differ appreciably in expression of TSA from the primary tumour, although slight quantitative and qualitative differences of TSA expression in primary and metastatic tumours might not be detected by the *in vitro* tube LAI assay.

Moreover, calculation of the mean non-adherence of leucocytes from groups of control subjects and patients with limited and metastatic breast cancer, indicated that the metastatic tumour was producing and shedding TSA into the circulation which coated *in vivo* LAI-reactive cells and caused the loss of their property of glass adherence when incubated *in vitro* with either specific or non-specific cancer extracts. By contrast, in patients with limited cancer, the circulating monocytes by and large have not yet encountered TSA, so that they have retained their glass adherence when incubated with non-specific antigens.

LAI reactivity to the tumour disappeared rapidly (2–4 months) after removal of the principal source of the antigenic stimulus, the primary cancer, even when some patients harboured distant micrometastases. The disappearance of a measurable antitumour immune response in the patients who had micrometastases possibly reflected a quantity of metastatic cancer that was too small to provide an adequate antigenic stimulus to the immune system. In Stage I breast cancer, many patients were LAI$^+$ when the tumour burden was small or even *in situ*, and hence the lack of LAI$^+$ in patients with micrometastases was not necessarily the result of an insensitive assay method. When the distant micrometastases grew, LAI of the host's circulating leucocytes again became detectable, suggesting that a certain minimum of tumour was required to stimulate the immune system. This quantity of tumour needed to trigger the immune response is not known. Many local recurrences are 1 cm in size and a 1cm tumour contains $10^9$ cancer cells. The presence of LAI reactivity some months before the tumour is detectable suggests that $10^5$–$10^6$ tumour cells may be required to stimulate the LAI response. Another possible explanation of the disappearance of antitumour immunity in patients after surgery who harbour micrometastases is that the cancer cells are in an immunologically privileged location.

The LAI results of the monitored patients suggests that the antitumour immunity of the host fades, and that the tumour-specific immune response ceases to play an active role in the rejection of micrometastases by 6 months after surgery. Not until the micrometastases have increased in cell numbers does the immune system return. Of course, other aspects of the tumour-specific immune response, not measured by tube LAI, could be active, and their failure may allow the tumour to escape and enter a rapid growth phase. Assessment of other aspects of the immune response to tumours by *in vitro* assays of microcytotoxicity, cytotoxicity and macrophage migration in human tumours have also detected, however, the waning of antitumour immunity after surgery (Anderson *et al.*, 1970; Bull *et al.*, 1973; Jones & Turnbull, 1974; Reiche *et al.*, 1976; Elias *et al.*, 1977; Canevari *et al.*, 1975; O'Toole *et al.*, 1973; Unsgaard & O'Toole, 1975).

In an animal model, Eccles & Alexander (1972) showed that the antitumour immune response expressed in the first month after surgery is critical in determining whether micrometastases are rejected or eventually grow. In human cancer the extent and duration of the transient immunosuppression produced by surgery (Grosser & Thomson, 1975; Marti & Thomson, 1976) and the vigour of the antitumour immune response in the month or so immediately after surgery, may prove to be critical factors that determine the fate of micrometastases.

In an animal tumour model, the host's tumour-specific immune response was detectable before TSA could be measured in the circulation (Thomson, 1975). Serological tests for the detection of tumour products in human sera, such as carcinoembryonic antigen (Thomson *et al.*, 1969) and alkaline phosphatase (Foti *et al.*, 1977), give the greatest values when the tumour burden is comparatively large. The present study suggests that the human host also expresses tumour-specific immunity when the tumour burden is small; thus, the early detection of human cancer by immunological assays will depend on the development of reliable methods to measure the antitumour immune response.

This work was supported by the Medical Research Council of Canada, the National Cancer Institute of Canada and the Montreal Cancer Research Society Inc.

We thank the members of the Department of Surgery for allowing us to study their patients, Dr W. Duguid, Pathologist-in-Chief, for materials and pathology reports and Dr P. Gold, Director of the Division of Clinical Immunology and Allergy, for helpful advice.

We appreciate the assistance of Dr A. B. Miller, Director, National Cancer Institute of Canada, for interpreting the statistical significance of the results in the various groups.

We thank Ms Mary Naughton for typing this manuscript.

### REFERENCES

ANDERSON, V., BJERRUM, O., BENDIXEN, J., SCHIODT, T. & DISSING, I. (1970) Effect of autologous mammary tumor extracts on human leukocyte migration *in vitro*. *Int. J. Cancer*, **5**, 357.

ARMITSTEAD, P. R. & GOWLAND, G. (1975) The leucocyte adherence inhibition test in cancer of the large bowel. *Br. J. Cancer*, **32**, 568.

BALDWIN, R. W. (1975) *In vitro* assays of cell-mediated immunity to human solid tumors: problems of quantitation, specificity and interpretation. *J. Natl Cancer Inst.*, **55**, 745.

BLACK, M. M., ZACHRAU, R. E., SHORE, B. & LEIS, H. P. (1976) Biological considerations of tumor-specific and virus-associated antigens of human breast cancers. *Cancer Res.*, **36**, 769.

BULL, D. M., LEIBACH, J. R., WILLIAMS, M. A. & HELMA, R. A. (1973) Immunity to colon cancer assessed by antigen induced inhibition of mixed mononuclear cell migration. *Science*, **181**, 957.

CANEVARI, S., FOSSATI, G., DELLA PORTA, G. & BALZARINI, C. P. (1975) Humoral cytotoxicity in melanoma patients and its correlation with the extent and course of the disease. *Int. J. Cancer*, **16**, 722.

DEICHMAN, G. I. & KLUCHAREVA, T. E. (1966) Loss of transplantation antigen in primary Simian Virus induced tumors and their metastases. *J. Natl Cancer Inst.*, **36**, 647.

ECCLES, S. A. & ALEXANDER, P. (1972) Role of macrophages in tumour immunity. I. Co-operation between macrophages and lymphoid cells in syngeneic tumour immunity. *Immunology*, **23**, 615.

ELIAS, E. G., ELIAS, L. L., DIDOLKAR, M. S. & HEBEL, J. R. (1977) Cellular immunity in patients with colorectal adenocarcinoma measured by autologous leukocyte migration inhibition. *Cancer*, **40**, 687.

FENYO, E. M., KLEIN, E., KLEIN, G. & SWIECH, K. (1968) Selection of an immunoresistant Moloney lymphoma subline with decreased concentration of tumor specific surface antigens. *J. Natl Cancer Inst.*, **40**, 69.

FLORES, M., MARTI, J. H., GROSSER, N., MACFARLANE, J. K. & THOMSON, D. M. P. (1977) An overview: antitumor immunity in breast cancer assayed by tube leukocyte adherence inhibition. *Cancer*, **39**, 494.

FOTI, A. G., COOPER, J. F., HERSCHMAN, H. & MALVAEZ, R. R. (1977) Detection of prostatic cancer by solid-phase radioimmunoassay of serum prostatic acid phosphatase. *N. Engl. J. Med.*, **297**, 1357.

FUJISAWA, T., WALDMAN, S. R. & YONEMATO, R. H. (1977) Leukocyte adherence inhibition by soluble tumor antigens in breast cancer patients. *Cancer*, **39**, 506.

GROSSER, N., MARTI, J. H., PROCTOR, J. W. & THOMSON, D. M. P. (1976) Tube leukocyte adherence inhibition assay for the detection of anti-tumour immunity: I. Monocyte is the reactive cell. *Int. J. Cancer*, **18**, 39.

GROSSER, N. & THOMSON, D. M. P. (1975) Cell-mediated antitumour immunity in breast cancer patients evaluated by antigen-induced leukocyte adherence inhibition in test tubes. *Cancer Res.*, **35**, 2571.

GROSSER, N. & THOMSON, D. M. P. (1976) Tube leukocyte (monocyte) adherence inhibition assay for the detection of anti-tumour immunity: III. "Blockade" of monocyte reactivity by excess free antigen and immune complexes in advanced cancer patients. *Int. J. Cancer*, **18**, 58.

HALLIDAY, W. S. & MILLER, S. (1972) Leukocyte adherence inhibition: a simple test for cell-mediated tumor immunity and serum blocking factors. *Int. J. Cancer*, **9**, 477.

HAYWOOD, G. R. & MCKHANN, C. F. (1971) Antigenic specificities on murine sarcoma cells. Reciprocal relationships between normal transplantation antigens (H-2) and tumor specific immunogenicity. *J. Exp. Med.*, **133**, 1171.

HELLSTRÖM, I., HELLSTRÖM, K. E. & SHANTZ, G. (1976) Demonstration of tumor-associated immunity with a leukocyte adherence inhibition (LAI) assay. *Int. J. Cancer*, **18**, 354.

HELLSTRÖM, I., HELLSTRÖM, K. E., VAN BELLE, G. & WARNER, G. A. (1977) Leukocyte-mediated reactivity of human tumors as detected by the leukocyte adherence inhibition test. I. Demonstration of tumor type-specific reaction. *Am. J. Clin. Pathol.*, **68**, 706.

HERBERMAN, R. B. & OLDHAM, R. K. (1975) Problems associated with study of cell-mediated immunity to human tumors by microcytotoxicity assays. *J. Natl Cancer Inst.*, **55**, 749.

HOLAN, V., HASEK, M., BUBENIK, J. & CHUTNO, J. (1974) Antigen-mediated macrophage adherence inhibition. *Cell. Immunol.*, **13**, 107.

HOLT, P. G., ROBERTS, L. M., FIMMEL, P. J. & KEAST, D. (1975) The LAI microtest: a rapid and sensitive procedure for the demonstration of cell-mediated immunity *in vitro*. *J. Immunol. Meth.*, **8**, 277.

JONES, B. M. & TURNBULL, A. R. (1974) *In vitro* cellular immunity in mammary carcinoma. *Br. J. Cancer*, **29**, 336.

LEVESON, S. H., HOWELL, J. H., HOLYOKE, E. D. & GOLDROSEN, M. H. (1977) Leukocyte adherence inhibition; an automated microassay demonstrating specific antigen recognition and blocking activity in two murine tumor systems. *J. Immunol. Meth.*, **17**, 153.

LOPEZ, M. J. & THOMSON, D. M. P. (1977) Isolation of breast cancer tumour antigen from serum and urine. *Int. J. Cancer*, **20**, 834.

LOWRY, O. H., ROSEBROUGH, N. J., FARR, A. L. & RANDALL, R. J. (1951) Protein measurements with the folin phenol reagent. *J. Biol. Chem.*, **193**, 265.

MALUISH, A. & HALLIDAY, W. J. (1974) Cell mediated immunity and specific serum factors in human cancer: the leukocyte adherence inhibition test. *J. Natl Cancer Inst.*, **52**, 1415.

MARTI, J. H., GROSSER, N. & THOMSON, D. M. P. (1976) Tube leukocyte adherence inhibition assay for the detection of anti-tumour immunity. II. Monocyte reacts with tumour antigen *via* cytophilic anti-tumour antibody. *Int. J. Cancer*, **18**, 48.

MARTI, J. & THOMSON, D. M. P. (1976) Anti-tumour immunity in malignant melanoma assay by tube leucocyte-adherence inhibition. *Br. J. Cancer*, **34**, 116.

O'TOOLE, C., UNSGAARD, B., ALMGARD, L. E. & JOHANSSON, B. (1973) The cellular immune response to carcinoma of the urinary bladder: correlation to clinical stage and treatment. *Br. J. Cancer*, **28**, 266.

POWELL, A. E., SLOSS, A. M., SMITH, R. N., MAKLEY, J. T. & HUBAY, C. E. (1975) Specific responsiveness of leukocytes to soluble extracts of human tumors. *Int. J. Cancer*, **16**, 905.

REICHE, K., ARNDT, A. & PASTERNAK, G. (1976) Cellular immunity in mammary cancer patients as measured by the leukocyte migration test—a follow-up study. *Int. J. Cancer*, **17**, 212.

RUTHERFORD, J. C., WALTERS, B. A. J., CAVAYE, G. & HALLIDAY, W. J. (1977) A modified leukocyte adherence inhibition test in the laboratory investigation of gastrointestinal cancer. *Int. J. Cancer*, **19**, 43.

THOMSON, D. M. P. (1975) Soluble tumour-specific antigen and its relationship to tumour growth. *Int. J. Cancer*, **15**, 1016.

THOMSON, D. M. P., GOLD, P., FREEDMAN, S. O. & SHUSTER, J. (1976) The isolation and characterization of tumor-specific antigens of rodent and human tumors. *Cancer Res.*, **36**, 3518.

THOMSON, D. M. P., KRUPEY, J., FREEDMAN, S. O. & GOLD, P. (1969) The radioimmunoassay of circulating carcinoembryonic antigens of the human digestive system. *Proc. Natl Acad. Sci. U.S.A.*, **64**, 161.

UNSGAARD, B. & O'TOOLE, C. (1975) The influence of tumour burden and therapy on cellular cytotoxicity responses in patients with ocular and skin melanoma. *Br. J. Cancer*, **31**, 301.